# Frequency and Functional Characterization of *RUNX1* Germline Variants in Myeloid Neoplasms

**DOI:** 10.1155/2023/4738660

**Published:** 2023-06-02

**Authors:** Nikolaj Juul Nitschke, Marwa Almosailleakh, Yiyuan Niu, Jakob Werner Hansen, Klas Raaschou-Jensen, Jakob Schmidt Jespersen, Marianne Tang Severinsen, Anne Stidsholt Roug, Morten Frödin, Joachim Lütken Weischenfeldt, Mette Klarskov Andersen, Kirsten Grønbæk

**Affiliations:** ^1^Department of Hematology, Rigshospitalet, Copenhagen, Denmark; ^2^Biotech Research and Innovation Centre (BRIC), Faculty of Health and Medical Sciences, University of Copenhagen, Copenhagen, Denmark; ^3^Department of Haematology, Zealand University Hospital, Roskilde, Denmark; ^4^Department of Haematology, Odense University Hospital, Odense, Denmark; ^5^Finsen Laboratory, Rigshospitalet, Copenhagen, Denmark; ^6^Department of Hematology, Aalborg University Hospital, Aalborg, Denmark; ^7^Department of Clinical Medicine, Aalborg University, Aalborg, Denmark; ^8^Department of Clinical Medicine, Aarhus University Hospital, Aarhus, Denmark; ^9^Department of Clinical Genetics, Rigshospitalet, Copenhagen, Denmark

## Abstract

Current estimates suggest that up to 10% of patients with myeloid neoplasms (MN) harbor variants associated with a germline predisposition. A pathogenic variant in the runt-related transcription factor 1 gene (*RUNX1*) is a frequent cause of germline predisposition to MN. *RUNX1* variants detected in tumor tissue at a VAF close to 50% are potentially germline and causative of *RUNX1* familial platelet disorder with associated myeloid malignancies. Previous studies have found germline *RUNX1* variants in 3% of patients with acute myeloid leukemia; however, the frequency of germline *RUNX1* variants in less advanced myeloid neoplasms has not been examined. We screened 590 patients suspected of MN, excluding myeloproliferative neoplasms, for germline variants in *RUNX1*. We found *RUNX1* variants in 83 patients (14%) by targeted sequencing of tumor tissue. In 40 patients (6.8%), the VAF of *RUNX1* was above 30%. In 32 of the 40 patients, skin biopsies were available and used for Sanger sequencing to assess the germline status. Two of the tested variants (6.3%) were confirmed as germline, and both variants were curated as variants of unknown significance. To further explore the pathogenicity of these variants, we implemented a novel CRISPR-Select functional genetic assay. The assay demonstrated a profound effect on proliferation in K562 cells for a known pathogenic variant but no effect for the two germline variants detected in the study. We therefore propose that both germline variants are classified as likely benign. In this study, we show that *RUNX1* germline variants are rare in Danish patients with MN and use a novel assay for functional classification of germline *RUNX1* variants.

## 1. Introduction

Current estimates suggest that 10% of patients with myeloid neoplasms (MN) harbor variants associated with germline predisposition [1, 2]. Germline mutations in the runt-related transcription factor 1 gene (*RUNX1*) were first described in 1999, as causing inherited thrombocytopenia with a propensity to develop acute myeloid leukemia (AML) [3]. It has since been established to be one of the most common causes of germline predisposition to MN [4]. *RUNX1* is a critical transcription factor essential for establishing definitive hematopoiesis, as shown in a mouse model where knockout of *RUNX1* was embryonically lethal due to CNS hemorrhage [5]. In adult hematopoiesis, *RUNX1* plays a critical role in lymphoid development, megakaryocytic differentiation, and platelet formation. MYH10 and MYL9 regulations by *RUNX1* as well as repression of KLF1 in megakaryocytic-erythroid progenitors have been suggested as pathways connecting *RUNX1* to platelet formation [6, 7]. Several mechanisms have linked *RUNX1* haploinsufficiency to oncogenesis including altered inflammatory signaling [8], reduced ribosomogenesis [9], and an attenuated p53-DNA damage response [10]. Germline mutations in *RUNX1* are causal for *RUNX1* family platelet disorder (*RUNX1*-FPD) associated with a ~45% lifetime risk of malignancy [11], a varying degree of thrombocytopenia, and a high risk of eczema. Pathogenic germline mutations are generally located in the RUNT homology domain responsible for DNA binding and dimerization with core binding factor-*β* with a resulting haploinsufficiency [12].

*RUNX1* mutations are identified in 10% of patients with AML and myelodysplastic syndrome (MDS) [13–15]. While most mutations are assumed to be acquired, growing evidence suggests that a substantial fraction are of germline origin. In patients with AML, Simon et al. showed that 30% of variants in *RUNX1* with a variant allele frequency (VAF) greater than 30% were germline [16]. Bąk et al. identified three patients with AML with germline *RUNX1* variants in a cohort of 100 patients [17], and Ernst et al. showed that 8% of patients with AML who achieved complete remission had a persistent *RUNX1* variant throughout baseline and after achieving complete remission [18]. Distinguishing between somatic mutations with a high VAF and germline mutations can be challenging but crucial as it impacts clinical management, e.g., hematopoietic stem cell donor selection and genetic counselling. The frequency of germline *RUNX1* mutations in preleukemic conditions, e.g., MDS and premalignant phenotypes such as clonal cytopenia of unknown significance (CCUS), remains unknown. In this paper, we uncover the germline status of *RUNX1* variants in patients with a spectrum of MN (ICUS, CCUS, MDS, chronic myelomonocytic leukemia (CMML), and AML) and examine factors associated with germline *RUNX1* variants. Furthermore, we present a novel technique based on CRISPR-Cas9 for the functional classification of *RUNX1* germline variants.

## 2. Methods

### 2.1. Patient Cohort and Material

This study is part of the Danish research program: the program for translational hematology (PTH), approved by the Danish National Ethics Committee. Patients referred with suspicion of MN or with relapse/progression of MN, excluding myeloproliferative neoplasms (MPN), were included in PTH between 2018 and 2021. On the first visit to a Danish hematological department, written consent was obtained, and a skin biopsy, peripheral blood samples, and a bone marrow aspirate were drawn. Skin biopsies were taken from the area of the skin above the posterior superior iliac spine and frozen dry at -80°C. We excluded patients from the study if the clinical work-up provided sound evidence of a nonmyeloid disease as the cause of the patient's symptoms, e.g., acute lymphoblastic leukemia.

### 2.2. Next-Generation Sequencing (NGS)

As part of their clinical workup, 450 patients were sequenced with one of the following amplicon-based platforms: the Ion AmpliSeq™ AML Research Panel covering 19 genes, Sophia Genetics™ Myeloid Panel covering 30 genes, and Ion Torrent™ Oncomine™ Myeloid Assay covering 72 genes. Filtering and curation of variants were done at the local departments of clinical genetics, pathology, or hematology. Information on variants was obtained from patient's electronic records along with clinical information. The indication for NGS is not specified on a national level but relies on the individual physician/department; therefore, the date of sequencing in some patients differed from the date of inclusion in the PTH study. Furthermore, 237 patients were sequenced as part of the PTH research program using DNA extracted from live frozen mononuclear cells separated with LeucoSep (Greiner Bio-One) from either peripheral blood or bone marrow aspirates drawn at inclusion. Sequencing was performed on an Illumina NextSeq 500 using a targeted sequencing panel (Illumina TruSeq Custom Amplicon, Illumina) covering 145 genes previously related to myeloid neoplasms. We used BWA [19] to align reads to GRCh38, Picard tools [20] to sort the reads and mark duplicates, and GATK tools [21] to recalibrate base quality scores. We then used VarDict [22], SNVer [23], and LoFreq [24] to call variants and Funcotator [21] for annotation. Variants with less than 4 reads, read depths of less than 200 or more than 3000, not assigned to chromosome 1-22 or X or Y, and VAFs of less than 2% were filtered out. The remaining variants were manually reviewed. In 98 patients, we had sequencing data from both the clinical workup and the PTH study.

All *RUNX1* variants were annotated to the *RUNX1c* transcript (NM_001754.4). *RUNX1* variants classified as benign or likely benign in ClinVar [25] were excluded from further analysis. *RUNX1* variants with VAF greater than or equal to 30% were classified as high VAF *RUNX1* variants (hVAF-*RUNX1*). For patients with sequencing data from multiple time points, only *RUNX1* variants with a consistent VAF greater than 30% were deemed hVAF-*RUNX1*. Variants with a VAF less than 30% after allogeneic hematopoietic stem cell transplantation (alloHSCT) were included for germline analysis. Patients with a known myeloid germline predisposition syndrome were also excluded for further analysis.

### 2.3. Germline DNA Extraction and Sanger Sequencing

Germline DNA was isolated from frozen skin biopsies using the DNeasy® Blood and Tissue Kit (Qiagen #69504), according to the manufacturer's protocol. Before isolation, bloody tissue was removed, and biopsies were washed in PBS (Gibco™ 10010023). Polymerase chain reaction (PCR) was performed with EconoTaq PLUS GREEN 2X Master Mix (Lucigen #30033-1) on a Veriti 96-Well Thermal Cycler (Applied Biosystems®) as per manufacturer's protocol. PCR conditions were used in initial denaturing for 1 min at 94°C, then 30 cycles of 94°C for 10 s, 60°C–62°C for 30 s, 72°C for 15 s, and a final post-PCR extension for 5 min at 72°C. Primers were designed using Primer3 to amplify exons 4-8, including intronic regions close to the exons. List of primer properties is available upon request. Bidirectional Sanger sequencing (Eurofins Genomics) was performed in PCR-amplified regions of *RUNX1*, depending on the location of the variant detected with NGS. Chromatograms were analyzed using the genome analysis server [26].

### 2.4. CRISPR-Select

We used a CRISPR-Select cell-based variant knock-in assay to functionally assess the pathogenicity of germline variants. As we have recently reported the principle of the method [27], we will only describe the setup briefly. K562 cells were cultured in Roswell Park Memorial Institute 1640 medium (ATCC #30-2001) supplemented with 10% (v/v) fetal bovine serum (Thermo Fisher Scientific #12389802) and penicillin-streptomycin (Gibco™ #15140122).

GuideRNAs (gRNAs) were designed for *S. pyogenes* Cas9 with the online software Benchling (https://benchling.com). The base pairs to be mutated were located as close as possible to the genomic cut site to increase knock-in efficiency and within the protospacer adjacent motif (PAM) or the 1-10 PAM proximal nucleotides within the gRNA target site for the mutations to effectively destroy the Cas9 target site. Single-stranded oligodeoxynucleotide (ssODN) repair templates encoding mutations to be knocked in were designed such that the synonymous WT´ control mutation was placed within the same codon as the variant of interest to promote knockin at similar frequencies. gRNAs were used in the form of crRNA:tracrRNA duplexes purchased from IDT™ and reconstituted in nuclease-free duplex buffer at 100 *μ*M. For ribonucleoprotein (RNP) generation, Alt-R *S. pyogenes* Cas9 Nuclease V3 from IDT™ (#1081059) was used. ssODN repair templates were purchased from IDT™ as unmodified Ultramer DNA oligonucleotides at 100 *μ*M in IDTE, pH 8.0.

Briefly, for a nucleofection of 10^6^ k562 cells, 500 pmoles each of crRNA and tracr-RNA were mixed and allowed to complex by incubation for 10 min at room temperature. Next, 96 pmoles of Cas9 proteins were mixed with the crRNA:tracrRNA duplexes and incubated for further 10 min. Next, cells were resuspended in 80 *μ*l of electroporation solution and added to RNPs and 500 pmol each of variant and WT′ ssODN. Finally, the cell suspension was transferred to a nucleocuvette and electroporated in a Lonza 4D-Nucleofector device using the T-003 program.

Genomic DNA was extracted on days 2 and 24 after nucleofection from an aliquot of the cell cultures using the Quick-DNA™ Miniprep kit (Zymo #D4069) as per the manufacturer's protocol. The genomic target site was amplified for NGS analysis using 100 ng of genomic DNA as a template and a two-round PCR [28]: Primer pairs for amplification of the target site in the first PCR were designed to anneal 40-120 nt outside the region covered by the ssODN repair template and to generate PCR products of 230-350 bps, using a Primer-BLAST3 from NCBI (https://www.ncbi.nlm.nih.gov/tools/primer-blast/). The second-round PCR and relevant primer design and PCR conditions were performed as described [27]. After mixing roughly equal amounts of second-round PCR products, the amplicon sequencing library was made using the MiSeq Reagent Kit v2 (Illumina #MS-102-2002) and finally sequenced in a MiSeq instrument from Illumina, according to the manufacturer's instructions. Sequencing depths ranged from 20,000-200,000 reads per sample. NGS data were analysed by the CRISPResso2 online tool using default settings (https://crispresso.pinellolab.partners.org/submission) [29].

### 2.5. Statistics

Statistical analysis included the calculation of odds ratios from two-by-two tables and differences between groups using Fisher's exact test. Platelet levels in different groups were analyzed using a linear model that adjusted for sex, age, and baseline diagnosis. Variant/WT' ratios were compared with a two-tailed paired *t*-test.*p* < 0.05 was considered significant. All statistical analyzes were performed in R using version 3.6.1.

## 3. Results

### 3.1. Frequency of Germline RUNX1 Variants

**Figure 1:**
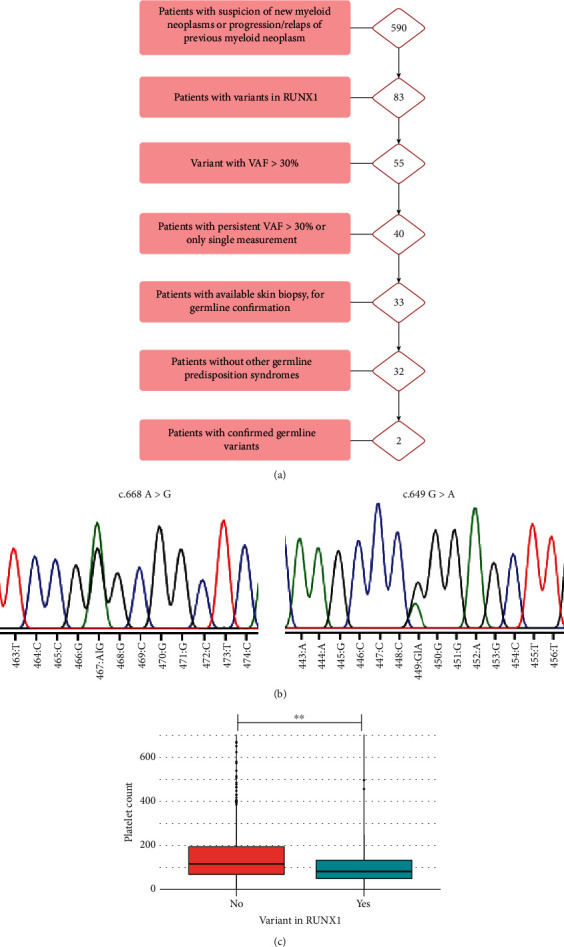
(a) Flow chart showing patient cohort. (b) Sanger chromatogram depicting two variants confirmed in germline tissue. (c) Platelet count in patients with/without RUNX1 variants. VAF: variant allele frequency.

In 590 unselected patients referred to a hematological department with suspected MN, excluding MPN, and without evidence of a nonmyleoid cause of symptoms, we identified a *RUNX1* variant in 83 patients (14%); see Figure 1(a). MDS was the most frequent diagnosis in our cohort, followed by AML and CCUS; see Supplemental Figure 1. CMML patients had the highest frequency of *RUNX1* variants including a high frequency of hVAF-*RUN1*; see Supplemental Figure 2. In 40 patients (6.8%) we found a hVAF-*RUNX1*. Of these, one patient was excluded due to a diagnosis of Shwachman-Diamond syndrome; 7 patients were excluded as no skin biopsy was available. In 2 of 32 patients (6.3%), the hVAF-*RUNX1* was confirmed as germline by bidirectional Sanger sequencing of DNA extracted from frozen skin biopsies; see Figure 1(b). None of the *RUNX1* variants with a VAF between 30 and 40% were germline. In our cohort, we observed a significant association between *RUNX1* variants and a lower platelet count (*p* = 0.003) after adjusting for age and diagnosis; see Figure 1(c); furthermore, patients with *RUNX1* variants had a significantly higher blast count; see Table 1. Clinical characteristics of patients with hVAF-*RUNX1* are provided in Supplemental Table 2.

### 3.2. Cooccurring Mutations

**Figure 2:**
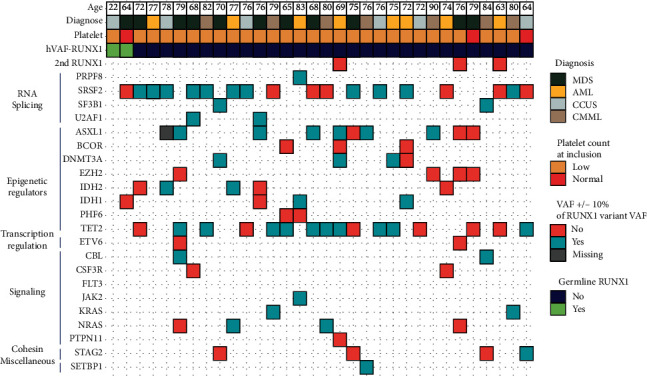
Cooccurring mutations in patients with high VAF RUNX1 variants. Each vertical line represents a patient. Patients with confirmed germline variants are sorted first. ^∗^*p* < 0.05; MDS: myelodysplastic syndrome; AML: acute myelogenous leukemia; CCUS: clonal cytopenia of unknown significance; CMML: chronic myelomonocytic leukemia; VAF: variant allele frequency.

**Figure 3:**
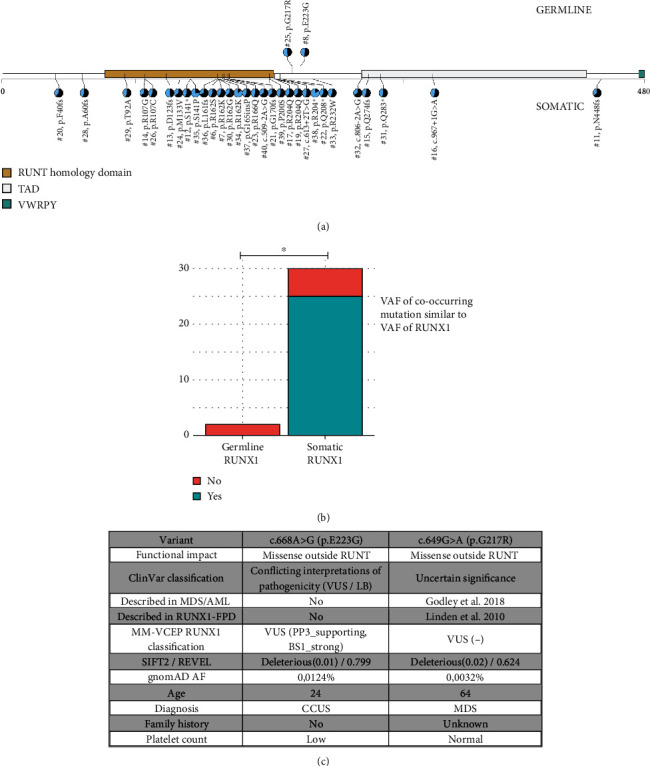
(a) Distribution of high VAF RUNX1 variants found in our cohort. Germline variants are depicted on top whereas somatic are depicted in the bottom. (b) Distribution of cooccurring mutations in patients with and without germline RUNX1 variants. ^∗^*p* < 0.05; MDS: myelodysplastic syndrome; AML: acute myelogenous leukemia; CCUS: clonal cytopenia of unknown significance; CMML: chronic myelomonocytic leukemia; VAF: variant allele frequency. (c) Curation of germline RUNX1 variants. MM-VCEP: Myeloid Malignancy Variant Curation Expert Panel; RUNX1-FPD: RUNX1 familial platelet disorder; gnomAD: Genome Aggregation Database; CCUS: clonal cytopenia of unknown significance; MDS: myelodysplastic syndrome.

In line with previous findings, we observed frequent cooccurring mutations in *SRSF2* (18/32), *TET2* (16/32), and *ASXL1* (10/22); see Figure 2(a). In patients with confirmed germline *RUNX1* variant, we did not observe any cooccurring mutations with a similar high VAF, whereas 5 out of 30 patients with somatic hVAF-*RUNX1* had cooccurring mutations at a similar VAF; see Figure 3(b).

### 3.3. Curation of Germline RUNX1 Variants

The hVAF-*RUNX1* were either frameshift, splice-site, nonsense, or missense, and the majority resided in the RUNT homology domain (RHD); see Figure 3(a). The germline variants (c.649G > A; p.G217R; and c.668A > G; p.E223G) were located between the Transactivation Domain (TAD) and the RHD.

The *RUNX1* variant p.E223G was identified in a 22-year-old woman with thrombocytopenia. Thrombocytopenia was diagnosed during an episode of deep vein thrombosis following a luxation of the right patella. The patient had no family history of hematological malignancies or thrombocytopenia. Hematological workup revealed no malignancy and only mild symptoms of bleeding (menorrhagia), and therefore she was initially diagnosed with CCUS due to the *RUNX1* variant. Subsequent examination revealed abnormalities of the aortic valve requiring surgery, periventricular nodular heterotopia detected with MR-cerebrum and a de novo missense VUS in the *FLNA* gene. Variants in *FLNA* are associated with periventricular nodular heterotopia, congenital heart disease, thrombocytopenia, among other things, and therefore, she was diagnosed with FLNA deficiency [30]. The *RUNX1* variant is classified as a variant with conflicting results in ClinVar (a report of VUS and likely benign). In gnomAD, it is identified at a frequency of 0.012%. In silico prediction with REVEL, using a recommended threshold of 0.75 from the ClinGen Myeloid Malignancy Variant Curation Expert Panel (MM-VCEP) [11], indicates a deleterious effect, score 0.799.

The p.G217R variant was identified in a 64-year-old male with MDS, a normal platelet count, and cooccurring mutations in *IDH1* and *SRSF2*, but family history was unavailable. The p.G217R variant is classified as a VUS in ClinVar and reported with a frequency of 0.003% in gnomAD. Using the same cutoff as mentioned above, REVEL does not predict the variant to be deleterious according to the recommended threshold, score 0.624. When applying ClinGen MM-VCEP *RUNX1*-specific curation, both variants are classified as VUS. See Figure 3(c) for curation details.

### 3.4. Cell-Based Functional Characterization of Germline RUNX1 Variants

**Figure 4:**
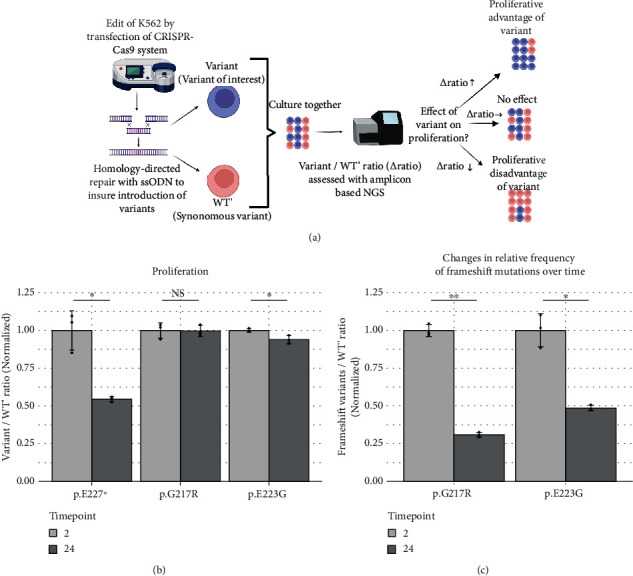
(a) Schematic overview of study design. (b) Ratio of variant (variant of interest) and WT' (synonymous variant) are plotted to estimate effect on proliferation. (c) Ratio of frameshift insertion and deletion to WT'. In (b) and (c), ratios have been normalized to the mean ratio at baseline (day 2). ^∗^*p* < 0.05. Assay was performed as triplicates; we plotted individual values and means ± the standard deviations.

To assess pathogenicity, we established CRISPR-Select for the functional analysis of *RUNX1* variants in relation to MN. As assay cells, we chose the human lymphoblast K562 cell line, which has previously been reported to express *RUNX1* [31]. As a CRISPR-Select readout for RUNX1 function, we chose proliferation, as Cai et al. [32] reported that loss of *RUNX1* was associated with decreased ribosome biogenesis and slow growth in HSPC. For each variant, we nucleofected a culture of K562 cells with CRISPR-Cas9 ribonucleoprotein targeting the variant genomic site along with two ssODN repair template coding for either the variant of interest or a synonymous control variant in the same codon, thereby producing a culture containing cells with the variant of interest (variant) and the corresponding control cells (WT´), see Figure 4(a). We examined the two germline *RUNX1* variants p.G217R and p.E223G in our cohort, and as a positive control, we used a known pathogenic variant in the same region, i.e., c.679G > T, p.Glu227Ter. To assess a relative change in Variant and WT´ cells over time as a readout for variant effect on cell proliferation and/or survival, we first extracted DNA from an aliquot of the cell populations at day two and day 24 after nucleofection. We then performed NGS on PCR products covering the region of knockin. As an outcome, we examined the variant and WT´ number of reads and compared the changes in the variant:WT´ ratio over time, using day two after nucleofection as the baseline. The pathogenic p.Glu227Ter control showed a large significant decrease in the variant:WT´ ratio at day 24, as depicted in Figure 4(b), revealing the expected loss of RUNX1 function. For the germline variant p.G217R, we observed no significant change over time, and for the germline variant p.E223G, we observed a significant change in variant:WT', but the mean difference over time was small compared to the known pathogenic control. The observed lack of effect could be a false negative due to a lack of selective pressure for various reasons in the specific experiments. However, the complete NGS characterization of CRISPR-Select editing outcomes allowed us to analyze the ratio of frameshift insertions and deletions (InDels) to WT´, which demonstrated strong negative selection against cells with frameshift InDels in the same cell culture dishes, where the variants exhibited no selective disadvantage; see Figure 4(c).

## 4. Discussion

Germline *RUNX1* variants have previously been examined in patients with AML, but the frequency of germline *RUNX1* mutations in pre-/less malignant phenotypes such as MDS and CCUS is unknown. Identification and classification of germline *RUNX1* variants is important as they have implications for clinical decision-making, e.g., donor selection. To estimate the frequency of germline *RUNX1* variants, we examined 590 patients and found that in 2 of 32 patients, hVAF-*RUNX1* was of germline origin, suggesting an absolute frequency of 0.3%. Previous studies reported absolute frequencies of germline *RUNX1* variants ranging from 1-3% in patients with AML, suggesting that the germline *RUNX1* frequency is higher in AML. In this study, we were not able to technically detect previously reported germline structural variants in *RUNX1* [33]. The two observed germline variants are classified as VUS, suggesting an even lower incidence of true disease-causing variants. In previous studies of germline *RUNX1* frequencies, no functional testing was performed [17, 34], potentially resulting in an overestimation of the frequency of disease-causing *RUNX1* variants. We had a relatively high frequency of *RUNX1* variants in our cohort compared to previous reports in MDS and AML [15, 35]; this could be due to the inclusion of CMML with a higher frequency of *RUNX1* variants.

Our data is real-world-based with variants being identified at multiple institutions using different sequencing tools, panels of genes, computational methods, and different approaches to variant curation. This makes the findings directly applicable to a clinical setting. However, there is a risk that variants are overlooked. To address this, we examined hVAF-*RUNX1* variants identified in patient samples which were sequenced using both our research panel and a clinical routine panel as part of the clinical workup (*n* = 98). Only two of ten hVAF-*RUNX1* were identified exclusively by the research panel (Supplemental Table 1). Unfortunately, no skin biopsy was available to examine the germline status of the c.e7-6TAAGC > C variant, but the c.1287_1341dup variant was confirmed as a somatic variant.

The pathogenicity of *RUNX1 variants* is difficult to assess. In silico predictions are unreliable, especially in RHD as this is a highly conserved region and most prediction tools rely on conservatism. The ClinGen Myeloid Malignancy Variant Curation Expert Panel has proposed criteria for classifying variants, but many variants remain classified as VUS. We designed a cell-based assay for RUNX1 variants, which determines the effect of CRISPR-Cas9-introduced variants on cell proliferation and/or survival. When introducing a known pathogenic variant, we observed an expected relative decrease in the proliferative capacity of variant cells compared to WT´ cells. With this assay, we were able to show that the two germline variants p.G217R and p.E223G had no functional effect in vitro on proliferative capacity, and we therefore reclassify them as likely benign variants. It should be emphasized that the CRISPR-Select format produces data of very high reliability. For instance, (i) CRISPR off-target actions and several other experimental artifacts are normalized out by the internal WT´ control, (ii) the data are based on hundreds of independent knockin cells for variant and WT´, (iii) results are based on analysis of the variant of interest in a proper genomic context without overexpression, and several additional features support the data reliability, as discussed by [27].

A previous study by Decker et al. used a set of three different assays to investigate the functional effect of *RUNX1* variants: heterodimerization ability with CBFB measured with a flow cytometry-based FRET assay, phosphorylation of *RUNX1* quantified with western blot and the ability of RUNX1 to activate transcription using a luciferase reporter assay [36]. Only one of the tested variants resided in the region where we detected two germline variants. Another study by Decker et al. analyzed different variants by applying a luciferase reporter assay to measure active transcription of three RUNX1 target genes, *CSF1R*, *ETV1*, and *MYL9* [37]. The assay was able to reclassify two VUSs as likely pathogenic variants but were only able to reclassify one out of four VUSs in the region where our two germline variants resided. In total, the two studies could reclassify two out of five variants in our region of interest, demonstrating the need for further functional assays such as the current. Furthermore, none of the known assays measure the impact of *RUNX1* variants on cell phenotypes, whereas our assay offers a direct way of testing whether a variant influences cell proliferation and/or survival. It is furthermore easy to modify the assay culture conditions with the aim of evaluating the effect of different exposures, e.g., drug screening or differentiation stimuli. A limitation of our assay is that we only used a truncating variant as a pathogenic control, but at this point, no known pathogenic missense variants have been reported in the region between RHD and TAD. Further studies are needed to investigate the performance of the assay in different regions of *RUNX1*.

## 5. Conclusions

Germline *RUNX1* variants are rare in this large Danish cohort of randomly sampled MN. In patients with a VAF greater than 30%, the frequency of germline variants was 6.3%. Here, we introduce a robust functional assay to evaluate *RUNX1* variants and were able to reclassify two *RUNX1* variants from VUS to likely benign.

## Figures and Tables

**Table 1:** Baseline characteristics of patients stratified according to *RUNX1* variants.

Variable	No	Yes	hVAF-RUNX1	*p*	Test
No.	508	42	40		
Diagnosis at baseline (%)				<0.001	
MDS	155 (30.5)	19 (45.2)	14 (35.0)		
AML	125 (24.6)	13 (31.0)	12 (30.0)		
CCUS	105 (20.7)	5 (11.9)	6 (15.0)		
CMML	32 (6.3)	5 (11.9)	8 (20.0)		
ICUS	91 (17.9)	0 (0.0)	0 (0.0)		
Age at inclusion (mean (SD))	69.0 (12.4)	71.8 (10.7)	71.9 (14.1)	0.189	
Sex = male (%)	326 (64.7)	22 (52.4)	31 (77.5)	0.059	
Platelet count at baseline [10^9^/L] (median [IQR])	116 [68, 194]	102 [49, 163]	73.50 [48, 103]	<0.001	Nonnorm
Hemoglobin at baseline (mmol/L) (median [IQR])	6.70 [5.70, 7.90]	6.45 [5.70, 7.22]	6.30 [5.47, 7.82]	0.423	Nonnorm
Leukocyte count at baseline (10^9^/L) (median [IQR])	4.60 [2.76, 7.84]	3.60 [2.33, 8.80]	4.66 [2.55, 9.45]	0.881	Nonnorm
LDH at baseline (U/L) (median [IQR])	210 [178, 260]	247 [177, 389]	227.00 [178, 281]	0.197	Nonnorm
Blast count, bone marrow (baseline) (median [IQR])	2.00 [0.00, 25.00]	10.00 [5.00, 41.00]	6.00 [1.25, 29.50]	0.001	Nonnorm

Baseline characteristics of patients in the study are divided into three groups: no RUNX1 variant, RUNX1 variant with a VAF < 30%, and RUNX1 variant with a VAF>30%. VAF: variant allele frequency; hVAF-RUNX1: RUNX1 variant with persistent VAF greater than 30%; MDS: myelodysplastic syndrome; AML: acute myelogenous leukemia; CCUS: clonal cytopenia of unknown significance; ICUS: idiopathic cytopenia of unknown significance; CMML: chronic myelomonocytic leukemia; SD: standard deviation; IQR: interquartile range.

## Data Availability

It is not possible to deposit the data in public repositories, since these data are considered sensitive personal data according to Danish law and the European Union General Data Protection Regulation (GDPR) and thus cannot be shared with third parties without prior approval. To access the data, please contact the corresponding author at kirsten.groenbaek@regionh.dk. Access can only be granted for research purposes and only if a data processor or data transfer agreement can be made in accordance with Danish and European law at the given time. The expected timeframe from response until access is granted is ~6 months.

## Supplementary Materials

Supplementary Materials Supplementary Figure 1: distribution of diagnosis in the cohort. Ranked from highest to lowest frequency. MDS: myelodysplastic syndrome; AML: acute myelogenous leukemia; CCUS: clonal cytopenia of unknown significance; CMML: chronic myelomonocytic leukemia. Supplementary Figure 2: proportion of patients with *RUNX1* variants among the different myeloid neoplasms included in the cohort. MDS: myelodysplastic syndrome; AML: acute myelogenous leukemia; CCUS: clonal cytopenia of unknown significance; CMML: chronic myelomonocytic leukemia. Supplementary Table 1: variants in RUNX1 with VAF greater than 30% in patients sequenced both in a clinical setting and as part of the PTH study. VAF: variant allele frequency; PTH: program for translational hematology. Supplementary Table 2: clinical characteristics of patients with hVAF-*RUNX1* variants. hVAF-RUNX1: *RUNX1* variants with persistent VAF greater than or equal to 30%; VAF: variant allele frequency.
