# Supplementary material for: Frequency and Functional Characterization of RUNX1 Germline Variants in Myeloid Neoplasms
Source: Hum Mutat. 2023 Jun 2;2023:4738660. doi: 10.1155/2023/4738660 (PMC11919008; doi:10.1155/2023/4738660)
Supplement: Supplementary Materials — Supplementary Figure 1: distribution of diagnosis in the cohort. Ranked from highest to lowest frequency. MDS: myelodysplastic syndrome; AML: acute myelogenous leukemia; CCUS: clonal cytopenia of unknown significance; CMML: chronic myelomonocytic leukemia. Supplementary Figure 2: proportion of patients with RUNX1 variants among the different myeloid neoplasms included in the cohort. MDS: myelodysplastic syndrome; AML: acute myelogenous leukemia; CCUS: clonal cytopenia of unknown significance; CMML: chronic myelomonocytic leukemia. Supplementary Table 1: variants in RUNX1 with VAF greater than 30% in patients sequenced both in a clinical setting and as part of the PTH study. VAF: variant allele frequency; PTH: program for translational hematology. Supplementary Table 2: clinical characteristics of patients with hVAF-RUNX1 variants. hVAF-RUNX1: RUNX1 variants with persistent VAF greater than or equal to 30%; VAF: variant allele frequency. [file 4738660.f1.docx]

Supplemental figure 1. Distribution of diagnosis in the cohort. Ranked from highest to lowest frequency. MDS = Myelodysplastic syndrome, AML = Acute myelogenous leukemia, CCUS = Clonal cytopenia of unknown significance, CMML = Chronic myelomonocytic leukemia


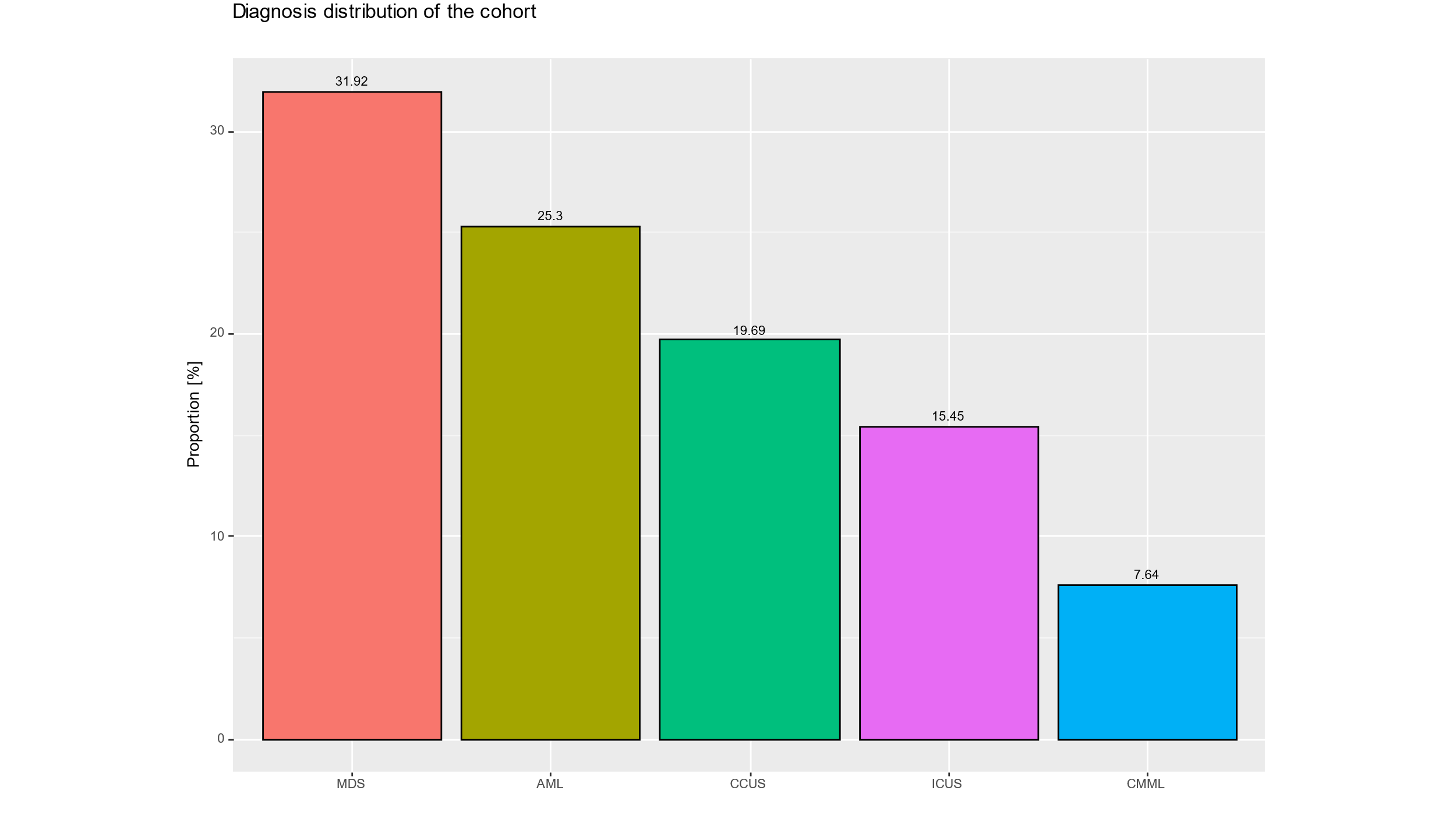


Supplemental figure 2. Proportion of patients with RUNX1 variants among the different myeloid neoplasms included in the cohort. MDS = Myelodysplastic syndrome, AML = Acute myelogenous leukemia, CCUS = Clonal cytopenia of unknown significance, CMML = Chronic myelomonocytic leukemia


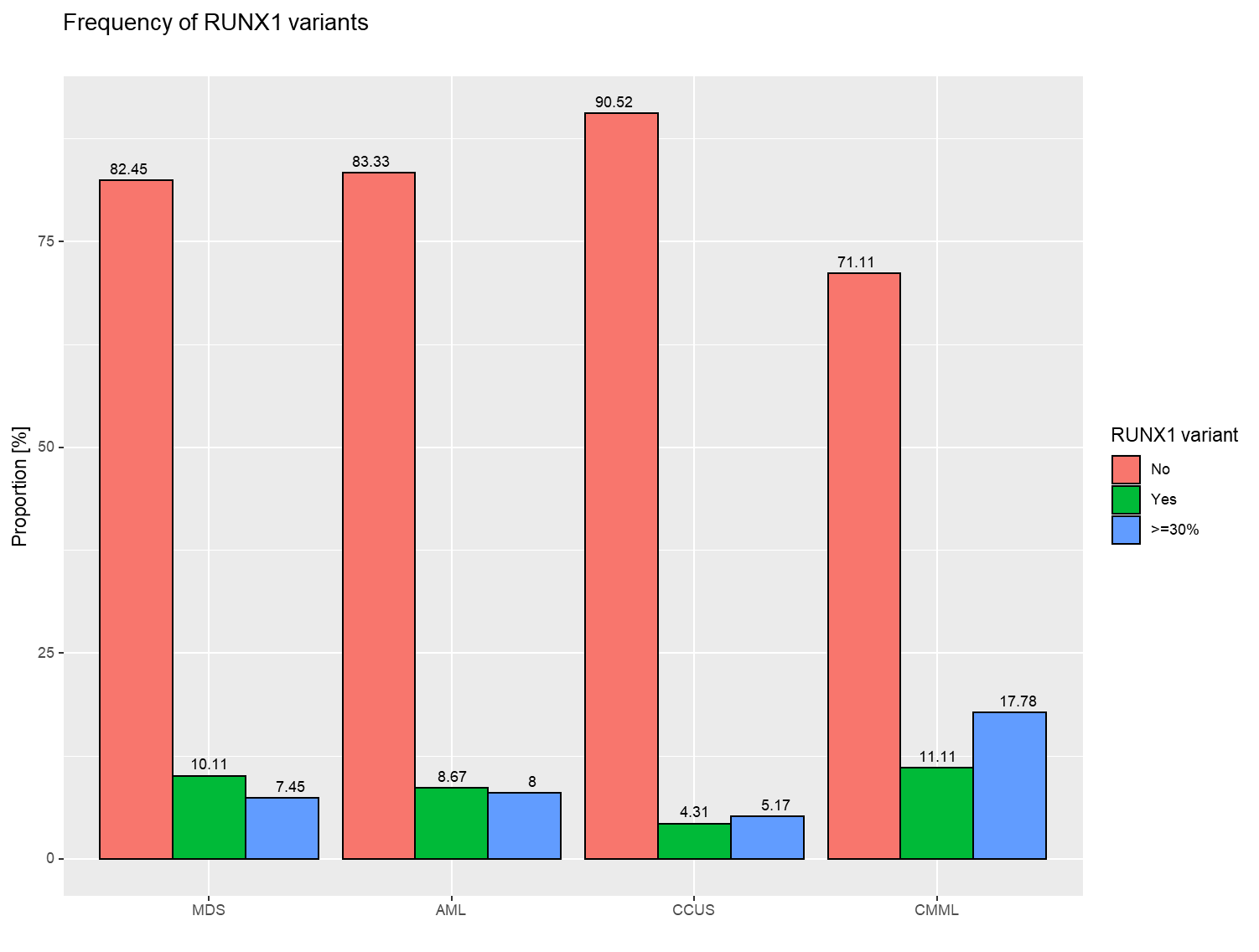


**Supplemental Table 1.** Variants in RUNX1 with VAF greater than 30% in patients sequenced both in a clinical setting and as part of the PTH study.

| **HGVSc** | **Clinical VAF** | **PTH VAF** | **Source** |
| --- | --- | --- | --- |
| NM_001754.4: c.951_952delTT | 40 | 45 | Clinical,PTH |
| NM_001754.4:c.319C>T | 31 | 40 | Clinical,PTH |
| NM_001754.4:c.e7-6TAAGC>C | NA | 41 | PTH |
| NM_001754.4:c.601C>T | 27 | 35 | Clinical,PTH |
| NM_001754.4:c.1145_1146insTGCC | 26 | 33 | Clinical,PTH |
| NM_001754.4:c.1302_1306delinsAAA | 36 | 36 | Clinical,PTH |
| NM_001754.4:c.274A>G | 36 | 47 | Clinical,PTH |
| NM_001754.4:c.790C>T | 5 | 42 | Clinical,PTH |
| NM_001754.4:c.30dup | 49 | 39 | Clinical, PTH |
| NM_001754.4:c.1287_1341dup | NA | 36 | PTH |

VAF = Variant allel frequency, PTH = Program for Translational Hematology

**Supplemental Table 2****.** Clinical characteristics of patients with hVAF-*RUNX1* variants.

| **ID #** | | **Platelet count at baseline [10^9/L]** | **History of bleeding** | **Family history of hematological disease** | **hVAF-*RUNX1* variant** | **Germline *RUNX1* variant** | **Co-occuring mutation** |
| --- | --- | --- | --- | --- | --- | --- | --- |
| 1 | | 154 | No | No | c.491_412insTG | No skin biopsy |  |
| 2 | | 61 | No | No | c.951_952delTT | No skin biopsy | *SRSF2, ASXL1* |
| 3 | | 66 | No | No | c.317G>T | No skin biopsy |  |
| 4 | | 56 | No | No | c.319C>T | No skin biopsy | *ETV6, BCOR, FLT3* |
| 5 | | 13 | No | No | c.485G>A | No skin biopsy | *JAK2* |
| 6 | | 59 | No | No | c.486G>T | Not germline | *TET2, SRSF2, IDH2* |
| 7 | | 79 | Unknown | Unknown | c.485G>A | Not germline |  |
| 8 | | 83 | Menoragi | No | c.668A>G | Germline |  |
| 9 | | 41 | Epistaxis | No | c.274A>G | No skin biopsy | *TET2, SRSF2* |
| 10 | | 103 | Gastro-intestinal bleeding | No | c.30_31insT | No skin biopsy | *TET2, SRSF2* |
| 11 | | 107 | No | No | c.1287_1341dup | Not germline | *SRSF2, IDH2, ASXL1* |
| 12 | | 48 | No | No | c.422C>A | Not germline | *NRAS, TET2, EZH2, CBL, ETV6, ASXL1* |
| 13 | | 68 | No | Mother:Blood disease (unspecific transfusion depend anemia) | c.367_368insG | Not germline | *SRSF2, U2AF1, CSF3R* |
| 14 | | 81 | Epistaxis, bruises | No | c.319C>G | Not germline | *TET2, SRSF2* |
| 15 | | 108 | No | No | c.820delC | Not germline | *DNMT3A, SF3B1, STAG2* |
| 16 | | 30 | No | No | c.967+1G>A | Not germline | *SRSF2, NRAS, IDH2* |
| 17 | | 87 | No | No | c.611G>A | Not germline | *SRSF2, TET2* |
| 18 | | 121 | No | Fathers father: Transfusion-depend anemia (unspecific) | c.523C>G | Not sequenced due to known Schwachman-Diamond disease |  |
| 19 | | 81 | Unknown | Unknown | c.611G>A | Not germline | *IDH1, IDH2, TP53, ASXL1, U2AF1* |
| 20 | | 98 | Excessive bleeding after operation | No | c.119_173del | Not germline | *KRAS, SRSF2, TET2* |
| 21 | | 68 | Unknown | Unknown | c.495_510dup | Not germline | *TET2, ZRSR2, BCOR, PHF6* |
| 22 | | 52 | No | No | c.622C>T | Not germline | *JAK2, IDH1, PHF6, PRPF8* |
| 23 | | 103 | Unknown | Unknown | c.497G>A | Not germline | *TET2, SRSF2, ASXL1* |
| 24 | | 44 | No | Mother, brother: Leukemia, unspecific | c.397A>G | Not germline | *NRAS, TET2, SRSF2* |
| 25 | | 151 | Unknown | Unknown | c.649G>A | Germline | *IDH1, SRSF2* |
| 26 | | 32 | No | No | c.319C>T | Not germline | *BCOR, DNMT3A, PTPN11, TET2, ASXL1* |
| 27 | | 102 | No | Brother: Myeolodysplastic syndrome | c.613+2T>G | Not germline | *SRSF2, ASXL1, TET2, STAG2* |
| 28 | | 142 | No | No | c.178dup | Not germline | *ASXL1, SETBP1* |
| 29 | | 84 | No | No | c.274A>G | Not germline | *SRSF2, TET2* |
| 30 | | 49 | Unknown | Unknown | c.484A>G | Not germline | *DNMT3A, TET2* |
| 31 | | 31 | No | No | c.847C>T | Not germline | *DNMT3A, IDH1, SRSF2, BCOR* |
| 32 | | 80 | No | No | c.806-2A>G | Not germline | *TET2, ZRSR2* |
| 33 | | 60 | Unknown | Unknown | c.694C>T | Not germline | *EZH2, ASXL1* |
| 34 | | 136 | Unknown | Unknown | c.485G>A | Not germline | *CSF3R, IDH2, SRSF2* |
| 35 | | 3 | No | No | c.421T>C | Not germline | *ASXL1, NRAS, EZH2, ETV6* |
| 36 | | 221 | No | No | c.481_482delCT | Not germline | *ASXL1, TET2, EZH2* |
| 37 | | 23 | No | No | c.496_497insCAT | Not germline | *SF3B1, CBL, STAG2* |
| 38 | | 20 | Unknown | Unknown | c.610C>T | Not germline | *SRSF2, TET2* |
| 39 | | 55 | No | No | c.598C>T | Not germline | *KRAS, SRSF2* |
| 40 | | 190 | No | No | c.509-2A>G | Not germline | *SRSF2, TET2, STAG2, ASXL1* |
|  | We were not able to retrieve data on history of bleeding and family history of hematological disease on all patients, which was noted as unknown. Genetic variants in *RUNX1* are annotated to NM_001754.5. hVAF-*RUNX1* = *RUNX1* variants with VAF greater than or equal to 30%, VAF = Variant allel frequency. | | | | | | |

**Institutional email addresses for submitting author**

**Department of Hematology, Rigshospitalet, Denmark**: [Nikolaj.juul.nitschke@regionh.dk](mailto:Nikolaj.juul.nitschke@regionh.dk),

Proof of employment <https://research.regionh.dk/da/persons/nikolaj-juul-nitschke>

**Biotech Research and Innovation Centre (BRIC), Faculty of Health and Medical Sciences, University of Copenhagen, Copenhagen, Denmark**: [Nikolaj.nitschke@bric.ku.dk](mailto:Nikolaj.nitschke@bric.ku.dk)
